# Assessing the Long-Term Effectiveness of Fractional CO_2_ Laser Treatment in Perimenopausal Women with Genitourinary Syndrome of Menopause—Single Center Preliminary Study

**DOI:** 10.3390/jcm14010242

**Published:** 2025-01-03

**Authors:** Sławomir Woźniak, Andrzej Woźniak

**Affiliations:** 1 3rd Chair and Department of Gynecology, Medical University of Lublin, Jaczewskiego 8, 20-954 Lublin, Poland; slavwo7572@gmail.com; 2 2nd Chair and Department of Gynecology, Medical University of Lublin, Jaczewskiego 8, 20-954 Lublin, Poland

**Keywords:** GSM, menopause, urogenital atrophy, CO_2_ laser

## Abstract

**Background:** Genitourinary Syndrome of Menopause (GSM) is a prevalent condition in postmenopausal women characterized by symptoms such as vaginal dryness, itching, and urinary tract issues due to declining estrogen levels. Despite its widespread impact on quality of life, GSM often remains underdiagnosed and without effective treatment. **Methods:** This study assessed the long-term efficacy of fractional CO_2_ laser treatment in alleviating GSM symptoms in perimenopausal women. The study involved 125 participants, with clinical evaluations conducted using vaginal pH, the Vaginal Health Index Score (VHIS), the Vaginal Maturation Index (VMI), and the Female Sexual Function Index (FSFI). **Results:** Results indicated significant improvements in these parameters, with pH levels decreasing, VHIS scores rising, and notable gains in VMI and FSFI observed up to 12 months post-treatment. This improvement has been validated through both subjective and objective assessments of GSM. **Conclusions:** The findings indicate that this method is effective and safe, with no significant side effects reported. However, conducting a long-term observational study on eventual longer protocol for maintaining the positive effect of this therapy should be conducted.

## 1. Introduction

Genitourinary Syndrome of Menopause (GSM) arises from declining estrogen levels during menopause, causing structural and functional changes in the genitourinary tract. After the menopause, the estrogen level decreases to below 30 pg/mL, causing symptoms that include vaginal dryness, itching, burning, dyspareunia, and lower urinary tract issues [1]. This phenomenon significantly impacts postmenopausal women’s quality of life, affecting physical comfort as well as emotional well-being [2]. GSM contributes to sexual health challenges, leading to a decline in sexual activity and life satisfaction. Extends to the urinary tract, causing urgency, frequency, and an increased risk of urinary tract infections [3,4].

According to the latest studies, about 50–70% of postmenopausal women report GSM symptoms [5]. Despite its prevalence, GSM is often an underdiagnosed condition. Diagnosis of GSM can be challenging due to varied symptoms, necessitating comprehensive assessments by healthcare providers. Addressing GSM’s significance involves raising awareness, promoting routine screenings, and encouraging proactive management to improve the understanding and management of this condition among postmenopausal women [6].

Various treatments, from hormone replacement therapy, which is currently the golden standard, to non-hormonal options, including energy-based devices (EBDs), aim to alleviate symptoms and improve quality of life. Innovative approaches like CO_2_ laser therapy show promise in addressing underlying physiological changes associated with GSM. Recognizing the symptoms, understanding the condition, and adopting comprehensive treatments are crucial for mitigating GSM’s impact on women’s health [7,8].

The use of CO_2_ laser in the treatment of genitourinary syndrome of menopause (GSM) has gained attention in recent years and is experiencing its renaissance. The treatment is minimally invasive and typically involves a series of sessions to achieve optimal results. The principle of the laser effect operates by directly influencing the vaginal mucosa, stimulating the metabolic activation of fibroblasts. The thermal impact of the laser initiates a reparative response, leading to the restructuring of vaginal tissue. This involves the generation of new collagen fibers and elastin within the atrophic epithelium. Simultaneously, the laser strengthens collagen structures in both the vulva and vagina, restoring tension to the vaginal muscles. By targeting epithelial cells, the laser releases energy, fostering epithelial maturation, thickening (resulting in an increased Vaginal Maturation Index), and exfoliation. Additionally, there is an observed rise in the formation of papillae within the epithelium and increased angiogenesis, enhancing the vascular network that supplies nutrients to the epithelium. The basal layer of the epithelium’s connective tissue sees an augmentation in collagen fiber quantity, restoring elasticity and resilience to mechanical injuries, akin to the premenopausal state [9,10,11].

In an increasing number of centers, therapies using EBD are commonplace. Lots of recent studies have shown promising outcomes in terms of symptom relief and improved quality of life for women experiencing genitourinary syndrome of menopause symptoms. Unfortunately, most of them are based on small groups, and the patient observation period is short. Therefore, the need for studies observing the long-term benefits of this therapy is crucial [12,13,14].

## 2. Materials & Methods

The research cohort initially comprised 159 women in the perimenopausal phase. Eligible participants were required to be (inclusion criteria): perimenopausal, experience at least one GSM symptom, and provide informed consent. Patients were excluded if they had (exclusion criteria): abnormal Pap smear results, active genital infections, recent use of systemic steroids or hormones (at least three months), pelvic organ prolapse (POP) > II or declined consent. Finally, this prospective study involved 125 participants aged 41 to 69, all of whom reported symptoms of Genitourinary Syndrome of Menopause (GSM). Following the first laser treatment, only 89 participants opted for a second session. Notably, all 36 individuals who discontinued treatment at this stage expressed satisfaction with the singular laser procedure. The entire laser therapy regimen was completed by 84 patients, forming the assessed study group.

The experiment aimed to assess the clinical efficacy of CO_2_ laser treatment for Genitourinary Syndrome of Menopause (GSM) symptoms in perimenopausal patients, specifically focusing on its long-term therapeutic effectiveness (within 12 months after the final laser treatment). The entire study received approval from the ethics committee. In accordance with the experimental protocol, each eligible patient for the therapy was required to undergo three sessions with a Monalisa Touch CO_2_ laser by DEKA, spaced at 6-week intervals. During each visit, a gynecological examination was conducted, assessing the severity of Urogenital Atrophy (UA) using the Vaginal Health Index Score (VHIS). The examination also included measuring vaginal pH, obtaining a cytological smear to determine Vaginal Maturation Index (VMI), and the completion of the FSFI questionnaire by the patient [15,16].

The follow-up program included 3 visits after finishing the treatment protocol: 1st follow-up visit—6 weeks after the last (3rd) treatment, 2nd follow-up visit—6 months after the last (3rd) treatment, and 3rd follow-up visit—12 months after the last (3rd) treatment. Final follow-up (12 months after 3rd treatment) was reached by 78 patients.

This study protocol offers a unique assessment of the contribution of this method in perimenopausal women with GSM, addressing the long-term durability of both objective and subjective outcomes beyond typical short-term studies. This multi-faceted approach allows for a more holistic understanding of how fractional CO_2_ laser treatment affects both physiological and quality-of-life outcomes. Additionally, with ongoing concerns about the risks of hormone replacement therapy, particularly in populations with contraindications, your study supports the urgent need for effective non-hormonal alternatives. 

## 3. Results

### 3.1. Vaginal pH Level

Before the therapy, the pH of the vaginal environment in the studied group of patients was 5.61 on average and ranged from 4.70 to 7.00 (Table 1). After each laser treatment, the pH level decreased significantly in relation to the condition before the treatment. The lowest pH level was achieved 6 weeks after the third treatment and averaged 4.69, ranging from 4.40 to 5.30.

6 months after the last (third) treatment, the pH increased to 4.84 but remained significantly lower than before the therapy. In a 12 month follow-up, the pH of the vaginal pH increased to 5.21 and was significantly higher than the level determined 6 months after the last treatment but remained lower than before the treatment.

### 3.2. Vaginal Health Index Score (VHIS)

The collective evaluation of VHIS among patients prior to therapy averaged 12.02, with a range from 7.00 to 16.00 (Table 2). The lowest VHIS scale results were observed for parameters such as epithelial mucous membrane (2.15) and vaginal secretions (2.17), followed by pH (2.39), vaginal hydration (2.60), and vaginal elasticity (2.70).

A total of 84 patients completed the entire series of three laser treatments. Six weeks following the last (3rd) laser treatment, the average VHIS for this women’s group was 21.50, with a range from 17.00 to 25.00. There was a notable enhancement in all VHIS parameters compared to their initial state.

6 months after the last (third) treatment, the overall VHIS score of this group of patients was on average 18.70 and ranged from 15.00 to 23.00.

12 months after the third procedure, the patients’ overall VHIS score was on average 15.01 and ranged from 11.00 to 21.00. There was still a statistically significant improvement in all parameters of vaginal well-being in relation to the condition before the procedure: the lowest result measured on the VHIS scale was determined for the vaginal secretion (2.74), and then for the following parameters: epithelial mucous membrane (2.86), vaginal hydration (2.95), pH (3.22), and the highest for the elasticity (3.24).

### 3.3. Vaginal Maturation Index (VMI)

The initial VMI of the patients averaged 21.5%, with a range from 11.5 to 32.0 (Table 3). Parabasal cells (P) constituted an average of 59.1%, intermediate cells (I) comprised 38.8%, and superficial cells (S) constituted only 2.1% on average, varying from 0.0 to 4.0%.

Before the first treatment, none of the 125 patients had an overall VMI score surpassing 49%, and the percentage of superficial cells (S) in all patients was below 5%.

Best values were achieved six weeks post the 3rd laser treatment. The average VMI outcome for this women’s group was 48.4%, ranging from 40.6 to 55.5. Parabasal cells (P) accounted for an average of 11.4%, intermediate cells (I) comprised 79.7%, and superficial cells (S) averaged 8.9%, varying from 7.0 to 11.0%. There was a notable enhancement in VMI, an increase in the count of superficial cells (S) and intermediate cells (I), and a reduction in the count of parabasal cells (P) in comparison to the state before the procedure. All patients in this group had a superficial cell count ≥5%.

Six months after the third procedure, the VMI result was on average 41.0% and ranged from 33.1 to 52.7.

Twelve months after the third procedure, the patients’ VMI score was on average 27.1% and ranged from 19.2 to 36.5. There was still a statistically significant increase in VMI and an increase in the percentage of superficial cells (S) and the intermediate cells (I) and a decrease in the percentage of parabasal cells (P) in relation to the condition before the procedure.

### 3.4. Female Sexual Function Index (FSFI)

The overall assessment of the FSFI in the study group before the therapy was on average 12.79 and ranged from 1.20 to 22.50 (Table 4). The lowest result was achieved by the patients in the domains of pain (1.81) and orgasm (1.91), and then in the domains of lubrication (2.03), arousal (2.30), desire (2.34), and satisfaction (2.39).

Six weeks after the third procedure, the overall FSFI score was the highest—24.39 and ranged from 20.90 to 30.30. There was a significant improvement in all the FSFI domains in comparison to the baseline. The lowest score measured on the FSFI scale was achieved by the patients in the domains of pain (3.94) and desire (3.97), followed by arousal (4.02), lubrication (4.05), and orgasm (4.05), and the highest for the satisfaction (4.35).

Six months after the third procedure, the general FSFI score in the study group was on average 21.88 and ranged from 17.20 to 27.90.

Twelve months after the third procedure, the general FSFI score of the group was on average 18.42 and ranged from 14.80 to 24.00. At this long-term follow-up, there was still a significant improvement in all the FSFI domains compared to the state before the treatment. The patient’s lowest score on the FSFI scale was achieved in the domains of desire (2.76) and pain (2.86), followed by lubrication (2.99), arousal (3.11), and orgasm (3.26), with the highest for satisfaction (3.44).

## 4. Discussion

The present study aimed to assess the long-term effectiveness of fractional CO_2_ laser treatment in perimenopausal women suffering from Genitourinary Syndrome of Menopause (GSM). Results demonstrate significant improvements in objective and subjective parameters, including vaginal pH, Vaginal Health Index Score (VHIS), Vaginal Maturation Index (VMI), and Female Sexual Function Index (FSFI), which are consistent with findings from previous studies, albeit with some variations in magnitude and duration of effect.

### 4.1. Vaginal pH

In the conducted study, the mean vaginal pH significantly decreased from 5.61 before treatment to 4.69 six weeks after the third treatment. This improvement persisted, although to a lesser extent, up to 12 months post-treatment, with a mean pH of 5.21. Comparatively, a study by Salvatore et al. (2015) reported a reduction in vaginal pH from 5.5 to 4.5 after CO_2_ laser treatment, with sustained improvements at 12 months follow-up [17]. Similarly, Filippini et al. (2017) observed a significant decrease in vaginal pH from 5.7 to 4.6, which remained stable for 12 months [18]. These findings corroborate our results, suggesting that fractional CO_2_ laser treatment effectively lowers vaginal pH, creating a more favorable vaginal environment and reducing GSM symptoms by its impact on epithelial structure and thus vaginal microflora. According to Takacs, Peter et al. (2020), laser treatment stimulates collagen synthesis by the fibroblasts, tissue remodeling, and increased epithelial thickness, all of which contribute to a microenvironment that supports healthier pH levels and may discourage the thinning of vaginal mucosa as well as the growth of pathogenic microflora associated with GSM symptoms [19]. Future studies could explore this connection, using microbiome analysis pre- and post-laser treatment to clarify the treatment’s effects on the vaginal flora and its potential in symptom relief.

### 4.2. Vaginal Health Index Score (VHIS)

A study showed a substantial increase in VHIS from a pre-treatment mean of 12.02 to 21.50 six weeks after the third treatment. At the 12-month follow-up, the VHIS decreased slightly to 15.01 but remained significantly higher than baseline. These results align with those of a randomized, double-blind, placebo-controlled study performed by Cruz et al. (2018), who reported an increase in VHIS from 13.5 to 20.8 six weeks post-treatment, maintaining an improved score of 17.2 at 12 months [20]. Another study by Tadir et al. (2017) demonstrated a rise in VHIS from 11.7 to 19.5 post-treatment, with sustained benefits observed up to one year [21]. These consistent findings across multiple studies underscore the efficacy of CO_2_ laser treatment in enhancing vaginal health and alleviating GSM symptoms over the long term. The laser’s ability to enhance tissue elasticity, hydration, and vascularity likely plays a key role in these improvements. CO_2_ laser therapy has been shown to increase collagen deposition and angiogenesis in vaginal tissues, which may result in enhanced tissue integrity and moisture retention, both of which are critical for reducing GSM symptoms [22]. This vascular improvement may also be associated with enhanced nutrient and oxygen delivery, supporting tissue health and symptom relief over the long-term [23]. Additional histological studies evaluating post-laser tissue changes would be valuable, offering insights into cellular and vascular changes associated with VHIS improvements and helping define optimal treatment intervals for sustained effects.

### 4.3. Vaginal Maturation Index (VMI)

The VMI in the study significantly improved from a baseline mean of 21.5% to 48.4% six weeks after the third treatment. Although there was a decline to 27.1% at the 12-month follow-up, it remained higher than the initial value. These results are comparable to those of Pagano et al. (2018), who found an increase in VMI from 22.0% to 47.0% post-treatment, with a slight reduction to 30.0% after one year [24]. Additionally, Gaspar et al. (2017) observed a rise in VMI from 20.3% to 45.6%, with sustained improvements at 12 months [25]. These improvements in VMI indicate enhanced epithelial maturation, likely resulting from the laser’s stimulation of basal cell activity and epithelial turnover [15]. Given that GSM is characterized by reduced epithelial thickness and cellular immaturity due to estrogen decline, these findings suggest CO_2_ laser treatment as a viable approach to induce epithelial repair independent of estrogen stimulation. A study conducted by Shingyochi et al. (2017) suggests that CO_2_ laser treatment may upregulate growth factors, fostering epithelial proliferation and maturation [26]. Furthermore, sustained VMI benefits at 12 months post-treatment suggest that fractional CO_2_ laser treatment may induce long-term remodeling effects on the vaginal epithelium. To fully understand the potential of CO_2_ laser therapy for GSM, future research should include molecular analysis of epithelial markers, such as epidermal growth factor (EGF) and transforming growth factor-beta (TGF-β), pre- and post-treatment. This would provide a clearer picture of how laser therapy affects cellular pathways involved in epithelial regeneration.

### 4.4. Female Sexual Function Index (FSFI)

Conducted studies are consistent with those reported in similar research, such as Salvatore et al. (2015), Pieralli et al. (2016), and Filippini et al. (2017) [17,18,27]. In the study, the baseline FSFI score was 12.79; after six weeks, the overall FSFI score improved significantly to 24.39, ranging from 20.90 to 30.30, at 12 month follow-up, the FSFI score averaged 18.42, ranging from 14.80 to 24.00, still showing significant improvement from baseline. Similarly, Salvatore et al. (2015) reported an increase from a baseline FSFI score of 14.5 to 26.4 at 12 weeks, which slightly declined to 23.8 at six months [17]. Pieralli et al. (2016) found that the FSFI score rose from 13.0 to 25.0 at three months, with a slight reduction to 23.0 at six months [27]. Filippini et al. (2017) reported an initial FSFI score of 12.8 that increased to 24.7 after three months, dropping to 20.5 at 12 months [18]. All these studies demonstrate a pattern of rapid improvement in FSFI scores within the first few months following treatment, with a gradual decrease over time but sustained benefits compared to baseline. Laser therapy’s positive impact on FSFI may be due to improved neuromuscular function, as increased collagen deposition and tissue hydration can enhance vaginal sensitivity and comfort during sexual activity [14]. Additionally, improvements in vaginal pH and microbiome may reduce infection risks, indirectly supporting sexual health and comfort. Given the FSFI score declines noted over time, future studies could investigate the optimal maintenance schedule for laser treatments. Regular booster sessions may help sustain FSFI improvements, contributing to longer-lasting benefits in sexual satisfaction and quality of life [28]. Consequently, this may clarify how CO_2_ laser therapy enhances sexual health beyond symptom relief, potentially offering new insights into GSM-related neurovascular impairment and its management through non-hormonal therapies.

### 4.5. Satisfaction Rate

A study by Perino et al. (2015) reported a 70% satisfaction rate among postmenopausal women undergoing CO_2_ laser treatment, similar to our 72% satisfaction rate at 12 months [29]. This consistency further validates the treatment’s efficacy. However, there are discrepancies in the duration of sustained benefits, with some studies, such as that by González Isaza et al. (2018), showing prolonged improvement up to 18 months, suggesting that individual patient factors and treatment protocols might influence long-term outcomes [16].

### 4.6. Limitations and Future Directions

While our study demonstrates promising results, it is not without limitations. The sample size, though adequate, is relatively small, and the follow-up period, while comprehensive, could be extended to assess longer-term effects. Furthermore, our study’s single-center design may limit the generalizability of the findings. Future multicenter studies with larger cohorts and extended follow-up periods are warranted to validate and expand upon our results.

## 5. Conclusions

The use of energy-based devices, such as fractional CO_2_ lasers, in treating GSM symptoms has shown positive effects on the sexual function of perimenopausal women over a one-year follow-up period. This improvement has been validated through both subjective and objective assessments of GSM symptom severity, including tools like the Vaginal Health Index Score (VHIS), Female Sexual Function Index (FSFI), and Vaginal Maturation Index (VMI). Moreover, the findings indicate that this method is safe and effective, with no significant side effects reported. However, conducting a long-term observational study on eventual longer protocol for maintaining the positive effect of this therapy should be conducted.

## Figures and Tables

**Table 1:** pH values of the vaginal environment during CO_2_ laser therapy.

Before Treatment ^a^ (*n* = 125)	After 3rd Treatment ^a^ (*n* = 84)	*p* ^b^	6 Months After 3rd Treatment ^a^ (*n* = 84)	*p* ^b^	*p* ^e^	12 Months After 3rd Treatment ^a^ (*n* = 78)	*p* ^b^	*p* ^e^	*p* ^f^
(*n* = 125)	(*n* = 84)		(*n* = 84)			(*n* = 78)			
5.61 ± 0.498	4.69 ± 0.208	****	4.84 ± 0.223	****	ns	5.21 ± 0.348	****	****	****

^a^—results are presented as average; ^b^ *p*—the statistical significance of differences between the condition before the procedure and the 3rd treatment, as well as 6 and 12 months after the 3rd treatment; ^e^ *p*—the statistical significance of differences between the condition after the 3rd treatment and 6 and 12 months after the 3rd treatment; ^f^ *p*—the statistical significance of differences between the condition 6 and 12 months after the 3rd treatment; ns—no statistical difference; ****—*p* < 0.0001.

**Table 2:** Assessment of VHIS after each stages of therapy.

VHIS Parameter	Before Treatment ^a^ (*n* = 125)	After 3rd Treatment ^a^ (*n* = 84)	*p* ^b^	6 Months After 3rd Treatment ^a^ (*n* = 84)	*p* ^b^	*p* ^e^	12 Months After 3rd Treatment ^a^ (*n* = 78)	*p* ^b^	*p* ^e^	*p* ^f^
Elasticity	2.70 ± 0.458	4.66	****	3.92	****	****	3.24	****	****	****
Vaginal secretion	2.17	3.92	****	3.44	****	****	2.74	****	****	****
pH	2.39	4.43	****	4.04	****	*	3.22	****	****	****
Epithelial mucous membrane	2.15	4.45	****	3.71	****	****	2.86	****	****	****
Vaginal hydration	2.60	4.05	****	3.60	****	****	2.95	***	****	****
VHIS (score)	12.02	21.50	****	18.70	****	****	15.01	****	****	****

^a^—results are presented as average; ^b^ *p*—the statistical significance of differences between the condition before the procedure and the 3rd treatment, as well as 6 and 12 months after the 3rd treatment; ^e^ *p*—the statistical significance of differences between the condition after the 3rd treatment and 6 and 12 months after the 3rd treatment; ^f^ *p*—the statistical significance of differences between the condition 6 and 12 months after the 3rd treatment; *—*p* < 0.05; ***—*p* < 0.001; ****—*p* < 0.0001.

**Table 3:** Assessment of VMI after each stages of therapy.

Cells Type (%)	Before Treatment ^a^ (*n* = 125)	After 3rd Treatment ^a^ (*n* = 84)	*p* ^b^	6 Months After 3rd Treatment ^a^ (*n* = 84)	*p* ^b^	*p* ^e^	12 Months After 3rd Treatment ^a^ (*n* = 78)	*p* ^b^	*p* ^e^	*p* ^f^
VMI	21.5	48.4	****	41.0	****	****	27.1	****	****	****
Parabasal cells	59.1	11.4	****	23.5	****	****	49.8	****	****	****
Intermediate cells	38.8	79.7	****	70.0	****	****	45.5	****	****	****
Superficial cells	2.1	8.9	****	6.5	****	****	4.7	****	****	****

^a^—results are presented as average; ^b^ *p*—the statistical significance of differences between the condition before the procedure and the 3rd treatment, as well as 6 and 12 months after the 3rd treatment; ^e^ *p*—the statistical significance of differences between the condition after the 3rd treatment and 6 and 12 months after the 3rd treatment; ^f^ *p*—the statistical significance of differences between the condition 6 and 12 months after the 3rd treatment; ****—*p* < 0.0001.

**Table 4:** Assessment of FSFI after each stages of therapy.

FSFI Domain	Before Treatment ^a^ (*n* = 125)	After 3rd Treatment ^a^ (*n* = 84)	*p* ^b^	6 Months After 3rd Treatment ^a^ (*n* = 84)	*p* ^b^	*p* ^e^	12 Months After 3rd Treatment ^a^ (*n* = 78)	*p* ^b^	*p* ^e^	*p* ^f^
Desire	2.34	3.97	****	3.47	****	****	2.76	****	****	****
Arousal	2.30	4.02	****	3.62	****	*	3.11	****	****	***
Lubrication	2.03	4.05	****	3.71	****	*	2.99	****	****	****
Orgasm	1.91	4.05	****	3.64	****	**	3.26	****	****	*
Satisfaction	2.39	4.35	****	4.01	****	*	3.44	****	****	****
Pain	1.81	3.94	****	3.42	****	****	2.86	****	****	****
General Score	12.79	24.39	****	21.88	****	***	18.42	****	****	****

^a^—results are presented as average; ^b^ *p*—the statistical significance of differences between the condition before the procedure and the 3rd treatment, as well as 6 and 12 months after the 3rd treatment; ^e^ *p*—the statistical significance of differences between the condition after the 3rd treatment and 6 and 12 months after the 3rd treatment; ^f^ *p*—the statistical significance of differences between the condition 6 and 12 months after the 3rd treatment; *—*p* < 0.05; **—*p* < 0.01; ***—*p* < 0.001; ****—*p* < 0.0001.

## Data Availability

The data used to support the findings of this study are included within the article.
